# Effect of reproductive status on foraging behavior and fecal glucocorticoid metabolite levels in wild bat-eared foxes (*Otocyon megalotis*)

**DOI:** 10.1093/jmammal/gyag011

**Published:** 2026-03-11

**Authors:** Aliza le Roux, Keafon R Jumbam, André Ganswindt

**Affiliations:** Department of Zoology and Entomology, University of the Free State-Qwaqwa, Private Bag X13, Phuthaditjhaba, Free State, 9866, South Africa; Department of Zoology and Entomology, University of the Free State-Qwaqwa, Private Bag X13, Phuthaditjhaba, Free State, 9866, South Africa; Department of Physiology, University of the Witwatersrand, Private Bag 3, Johannesburg, Gauteng, 2050, South Africa; Department of Zoology and Entomology, Mammal Research Institute, University of Pretoria, Private Bag X20, Hatfield, Gauteng, 0028, South Africa

**Keywords:** fecal glucocorticoid metabolite concentrations, foraging rate, parental care, reproductive status, seasonal change

## Abstract

Diet may be fundamental to the extensive paternal care and reduced maternal care seen in bat-eared foxes (*Otocyon megalotis*). This termite-specialist would struggle to increase its energy intake by hunting large prey or provisioning such items to mates or pups. Consequently, lactating, physiologically challenged females need to invest more time in foraging, while males spend time with pups. However, there is little empirical evidence of the impacts of parental care on foraging behavior and stress-related hormone levels in free-living bat-eared foxes. We studied foraging behavior in 20 wild bat-eared foxes for 2 years, investigating how foraging behavior and fecal glucocorticoid metabolite (fGCM) levels varied with austral season in the study population. Thereafter, we evaluated how parental status may affect foraging rates, food sizes consumed, and fGCM levels as a proxy for physiological stress. We examined these changes in parents (*n *= 3) and non-parents (*n *= 17) as seasonal “activity” changed—that is, breeding season (pregnant phase), denning season (pup-rearing and guarding phase), and non-breeding season (independent adult phase). Small item consumption patterns mirrored overall foraging rates, which were lowest for all foxes in winter. Males increased foraging rates in spring, while all individuals ate more large items in summer. Mean fGCM levels in the population (0.41 µg g^−1^ organic content) were not affected by sex or austral season, but changed with seasonal activities, for parents in particular: parents had significantly lower fGCM levels in the breeding season. This may reflect reduced stress in these foxes, who successfully paired and increased their within-family socialization during the breeding season. Our findings suggest that behavioral adaptations, including foraging adaptations, are sufficient for bat-eared foxes to meet the physiological challenges of parenting. This is the first study to start unravelling the relationship between foraging rates, parenting behavior, and stress-related hormone levels in the Bat-eared Fox.

Like many canids, bat-eared foxes (*Otocyon megalotis*) are monogamous and live in small family groups (Pauw 2000; Wright et al. 2010). Unlike most other canids, this species is a dietary specialist, feeding predominantly on termites and small items (Berry 1981; Nel 1984; Stuart et al. 2003), which appears to have had significant consequences for parental care (McNab 1984; Oftedal and Gittleman 1989; Pauw 2000). Due to this dietary restriction, male bat-eared foxes cannot provision lactating females with large food items, and such provisioning (typical of paternal care in other canids) has only rarely been documented (Pauw 2000, Wright 2006; le Roux et al. 2014). Furthermore, female bat-eared foxes are expected to be ­distinctively challenged due to the demands of pregnancy and lactation (Oftedal and Gittleman 1989). In both domestic and wild carnivores, lactation places high energy demands on females, often increasing stress-related physiological responses and decreasing body condition (Scantlebury et al. 2002; Alekseeva et al. 2020). Female bat-eared foxes cannot readily increase their energetic intake by hunting larger food items and are therefore forced to spend considerably longer foraging periods on their small-bodied primary prey base, that is, termites. This leads to the prevalence of heavy male investment in paternal care (Maas and MacDonald 2004), while the primary contribution of the mother to parental care is nursing. Indeed, it is presumed that the extensive maternal nursing periods coupled with her nutritional needs could be driving allonursing and polygamous breeding groups within this species (Pauw 2000).

Despite the putative centrality of diet in the social structure and unusual paternal care patterns of bat-eared foxes, we know little about changes in foraging behavior in successful breeders. Most studies of parental care in wild bat-eared foxes have focused on time spent with pups (Maas and MacDonald 2004; Wright 2006) or a description of limited instances of provisioning (le Roux et al. 2014) and foraging innovation (Jacobs and Le Roux 2016), presumably driven by the physiological demands of parenting. Previous work suggests that social stress may increase fecal glucocorticoid metabolite (fGCM) concentrations in captive bat-eared foxes (le Roux et al. 2016). However, it is not clear how parenting and/or breeding would impact foraging and physiological stress in this monogamous canid. Indeed, there is still a significant lack of information on the behavioral endocrinology of parenting in wild carnivores in general, particularly for fathers who contribute to caring (de Bruin et al. 2016). While acknowledging that the relationship between stressors and glucocorticoids in wild animals remains unpredictable (Romero and Beattie 2022), we anticipated that the unique stressor of parenting would have a distinct impact on fGCM in parental bat-eared foxes compared with individuals who did not mate and breed successfully.

Our study aim was to assess how parenting might affect foraging behavior and fGCM concentrations, as one stress-related physiological biomarker, in wild bat-eared foxes. Importantly, seasonal changes may affect all individuals in a population, regardless of parental status: the austral season typically alters glucocorticoid secretion in wild mammals (Borniger et al. 2017), and bat-eared foxes are known to decrease foraging time in winter (Lourens and Nel 1990). We therefore first examined the impact of austral season on both foraging behavior and fGCM concentrations, regardless of parental status. We predicted that: (A) winter would be characterized by higher fGCM levels, for example, as in common marmosets (*Callithrix jacchus*; Garber et al. 2020), and American pikas (*Ochotona princeps*; Whipple et al. 2021) and lower foraging rates for all adult foxes; that (B) parents would increase energetic intake during pup dependence by elevating foraging rates and selecting for larger prey items; and (C) parents would exhibit heightened fGCM levels during this pup-rearing phase. Due to the additional energetic demands of lactation, we expected that mothers would exhibit a stronger response in (B) and (C) than would fathers.

## Methods

### Study location and population

We conducted this study on 20 habituated adult foxes (9 females, including 1 parent, and 11 males, including 2 parents) at Kuruman River Reserve (KRR, 28°59′S, 21°49′E), which covers an area of 32 km^2^ in the Northern Cape province of South Africa. The reserve consists of Kalahari Thornveld and perennial grasses on sandy dunes (Clutton-Brock et al. 1999) and experiences a mean annual rainfall of 282 mm, with temperatures ranging from –4.60 °C to 41.0 °C (Welch et al. 2017). We defined austral seasons as summer (December-February), autumn (March-May), winter (June-August), and spring (September-November). We further described 3 categories of seasonal “activity” (which aligned with austral seasonal changes to some extent) as breeding season (November-February), denning season (March-June), and non-breeding season (July-October). These activities symbolized periods when individuals were expectant/pregnant (breeding season); rearing pups, that is, parents chaperoning pups on foraging trips and protecting the den (denning season); and when pups became independent (non-breeding season).

### Foraging behavior recordings

We habituated animals and recorded their foraging behavior on Android tablets programmed with Cybertracker software as described in Jumbam et al. (2019). A “follow” session was defined as an observation period in which trained observers would locate a habituated fox and follow it on foot for a maximum of 2 h, starting at dusk when these nocturnal animals were most active. Focal animals were within 2-5 m from the observer, making it possible to record their social interactions and foraging behaviors on a tablet for the duration of the follow session.

### Fecal sample collection

During animal follows, we collected fecal samples within minutes of defecation (*n*  _males_ = 203, *n*  _females_ = 129), labelled them according to individual identity and date of collection, and immediately placed the samples on ice blocks in cooler bags for the duration of the follow. Upon arrival at the research base, we immediately transferred samples to temperatures of –20 °C until further processing at Endocrine Research Laboratory (ERL), University of Pretoria, South Africa. Due to the short duration of follows (2 h), we did not expect that the cool storage time before samples were frozen would lead to any alteration of fGCM concentrations (see also Osburn et al. 2025).

### Fecal glucocorticoid metabolite extraction and quantification

We lyophilized, pulverized, and sieved frozen fecal samples using a fine mesh to remove fibrous material. We extracted a weighed amount (0.10-0.11 g) of fecal powder by adding 3 ml of 80% ethanol in water and vortexed for 15 min, and centrifuged the suspension at 1,500 × *g* for 10 min. We transferred the formed supernatant into microcentrifuge tubes and stored it at –20 °C for further analysis.

A previous study validated a cortisol EIA for determining changes in fGCM levels in bat-eared foxes (le Roux et al. 2016), which we used to quantify fGCM concentrations in this study. Palme and Möstl (1997) provide the detailed assay characteristics, including a full description of assay components and cross-reactivities. Serial dilutions of fecal extracts gave displacement curves parallel to the standard curve (relative variation of the slope of the trend lines was *<*5%). The sensitivity of the EIA was 0.3 ng g^−1 ^dry weight. Intra- and inter-assay coefficients of variation, determined by repeated measurements of high and low value quality controls were 4.20% and 4.70%, as well as 11.1% and 14.1%, respectively.

### Determination of organic content and analyses

We accounted for organic content in feces due to high mineral content from accidental soil ingestion, which could impact the determination of hormone metabolite concentrations (de Bruin et al. 2018). We determined organic content by oven-drying the extracted fecal powder sediments at 70 °C. We weighed and incinerated the oven-dried samples at 430 °C for 1 h before placing them into a desiccator for 2 h. We then re-weighed samples and calculated the organic content as the difference in mass before and after incineration.

### Data analysis

We collected 57 ± 29 h (mean ± SD) observation time per individual (Table 1; Supplementary Data SD1). For each follow, we calculated foraging rates by dividing the sum of all ingested food items by the duration (in minutes) of the follow (i.e., items per min; Supplementary Data SD2). Additionally, we grouped food items into small (≤2 cm), medium (2-4.9 cm), and large (5-15 cm; Table 2).

**Table 1:** Number of observations (nightly follows) conducted on 20 bat-eared foxes, across seasonal activities.

Parental status	Breeding	Denning	Non-breeding
**Non-parent**	299	114	42
**Parent**	77	29	14

**Table 2:** Food size classification of the Bat-eared Fox diet.

Food size category	Measurement	Order or class^a^
**Small**	≤2 cm	Isoptera (termites) Hymenoptera (ants) Plant seeds Orthoptera (crickets)
**Medium**	2-4.9 cm	Neuroptera (antlions) Coleoptera (beetles) Lepidoptera (caterpillars) Orthoptera (grasshoppers) Mantodea (mantises) Hemiptera (cicadas) Lepidoptera (moths) Arachnida^a^ (spiders)
**Large**	5-15 cm	Rodentia (rodents) Diplopoda (millipedes) Lagomorpha (springhares) Reptilia^a^ (reptiles) Scorpiones (scorpions) Amphibia^a^ (frogs) Other (fungi)

Due to the infrequent consumption of large items by foxes, we combined medium and large food sizes into 1 category termed large items (Supplementary Data SD3). A non-significant number (0.0001) was added across all datasets to neutralize any zeroes still present in the dataset, even after this merger. For all models described below, we checked model assumptions and removed interactions that resulted in overfitted models.

We used generalized linear mixed effect models (GLMMs) using the “package lme4” in R (R Core Team 2024, version 4.3.2) to assess how foraging rates may be affected by austral season, sex, parental status—parents (*n *= 3) and non-parents (*n *= 17)—and seasonal activity. In all models described below, we controlled for individual identity as a random variable, and chose the final model based on the lowest Akaike Information Criterion (AIC). We log-transformed data and assessed the relationship between the fixed effects of sex, austral season, parental status, and seasonal activity with foraging rates, running separate models with the following dependent variables: the log of (i) overall foraging rates; (ii) foraging rates for small items; and (iii) foraging rates for large items. Because the relatively small dataset may reduce the power of statistical analyses by including many fixed effects, we did not incorporate all the fixed effects into 1 model but examined each fixed effect singly while controlling for individual identity. We also explored a limited set of interactions that could have biological salience, that is, the interaction between sex and parental status, between sex and austral season, and between parental status and seasonal activity. We presented only the 3 final models with the most explanatory power (based on AIC), per output variable.

We determined fGCM concentrations of 332 samples (Supplementary Data SD4), expressed per gram organic mass (µg/g organic content). To evaluate the effect of parental status on seasonal activity, we used GLMMs in R to assess the interactions of fixed effects with fGCM concentration (the dependent variable), while setting individual identity as a random variable. We used the same model structures and fixed effects as for the foraging data, examining 1 fixed effect per model, as well as the biologically relevant interactions in 3 additional models. We log-transformed the fGCM data to meet normality assumptions and chose the final model based on the lowest AIC.

## Results

### Variation in foraging rates

**Fig. 1:**
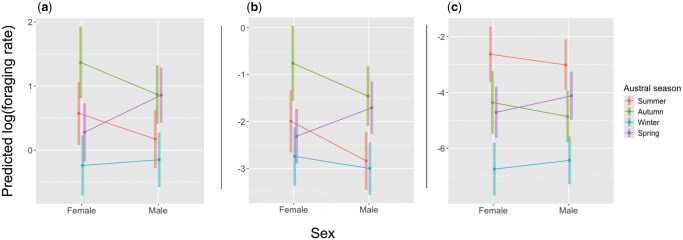
Predicted log of foraging rates (mean ± 95% CI) from generalized linear mixed effect models examining the link between austral season, sex, and the consumption of a) all items, b) small items, and c) large items by bat-eared foxes.

Foraging rates were lowest in winter for all items, small items, and large items (Table 3; Fig. 1), with the interaction between sex and austral season being the most significant predictor of bat-eared fox foraging rates (Table 4). In line with our predictions, foraging rates were lowest in winter for all output variables. Autumn was the season in which overall foraging rates were the highest, but for males, the high autumn consumption rates were matched by high consumption rates in spring (Fig. 1b). This may be ascribed to a significant rise in their consumption of small items in particular: whereas males generally consumed fewer small prey than did females, this pattern changed significantly in spring. Large items were consumed at the highest rates during summer for both sexes. The variation in foraging behavior of foxes across seasonal activities qualitatively matched these patterns.

**Table 3:** Daily foraging rates and fecal glucocorticoid metabolite (fGCM) values for bat-eared foxes at the Kalahari Research Centre.

Parental status	Seasonal activity	Sex	Foraging rate (items/minute)	*N*_foraging rate_	fGCM (µg/g organic content)	*N*_fGCM_
**Non-parent**	Breeding	Female	3.14 ± 3.578	154	0.417 ± 0.559	39
		Male	3.175 ± 4.453	145	0.398 ± 0.572	45
	Denning	Female	7.418 ± 5.833	41	0.726 ± 0.769	20
		Male	4.279 ± 4.907	73	0.469 ± 0.738	51
	Non-breeding	Female	1.296 ± 2.31	19	0.259 ± 0.212	45
		Male	2.238 ± 3.635	23	0.351 ± 0.371	68
**Parent**	Breeding	Female	2.054 ± 3.037	25	0.148 ± 0.060	6
		Male	6.169 ± 6.105	52	0.369 ± 1.051	16
	Denning	Female	4.125 ± 4.863	13	0.594 ± 0.746	9
		Male	8.498 ± 7.241	16	0.635 ± 0.506	13
	Non-breeding	Female	3.097 ± 3.47	5	0.369 ± 0.393	10
		Male	1.444 ± 1.436	9	0.408 ± 0.555	10

These data were log-transformed prior to statistical analyses. Sample sizes reflect the number of daily datapoints, not the number of individuals.

### fGCM variation

**Fig. 2:**
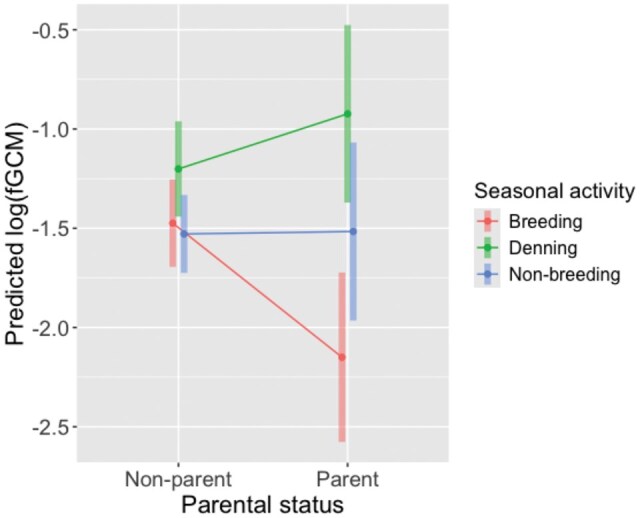
Predicted log of fecal glucocorticoid metabolites (fGCM, mean ± 95% CI) from a generalized linear mixed effect model, assessing the relationship between parental status, seasonal activity, and fGCM.

The most parsimonious model explaining variation in fGCM contained the interaction between parental status and seasonal activity as fixed effects. Whereas fGCM remained steady between seasonal activities, fGCM of parents were significantly lower than those of non-parental foxes (Table 5). This difference could be ascribed to a steep drop in fGCM in parents during the breeding season, specifically (Fig. 2).

**Table 4:** Model outputs from linear mixed effect models investigating daily foraging rates in bat-eared foxes.

Foraging rate: all items (df = 571)						
**Parameter**	**Coefficient**	**SE**	**CI_low**	**CI_high**	***t***	***P***
**(Intercept)**	0.573	0.243	0.096	1.05	2.361	0.019
***Austral season (autumn)***	***0.796***	***0.274***	***0.259***	***1.333***	***2.909***	***0.004***
***Austral season (winter)***	***–0.811***	***0.233***	***–1.268***	***–0.354***	***–3.486***	***0.001***
**Austral season (spring)**	*–*0.294	0.224	*–*0.733	0.146	*–*1.311	0.190
**Sex (male)**	*–*0.400	0.331	*–*1.05	0.249	*–*1.21	0.227
**Austral season (autumn): Sex (male)**	*–*0.101	0.367	*–*0.821	0.62	*–*0.275	0.784
**Austral season (winter): Sex (male)**	0.489	0.318	*–*0.136	1.114	1.536	0.125
***Austral season (spring): Sex (male)***	***0.98***	***0.309***	***0.374***	***1.586***	***3.175***	***0.002***
**Foraging rate: small items (df = 567)**						
**(Intercept)**	*–*1.99	0.332	*–*2.641	*–*1.339	*–*6.002	<0.001
***Austral season (autumn)***	***1.233***	***0.469***	***0.313***	***2.154***	***2.631***	***0.009***
**Austral season (winter)**	*–*0.75	0.404	*–*1.544	0.044	*–*1.856	0.064
**Austral season (spring)**	*–*0.321	0.386	*–*1.08	0.438	*–*0.831	0.406
**Sex (male)**	*–*0.846	0.453	*–*1.735	0.043	*–*1.869	0.062
**Austral season (autumn): Sex (male)**	0.147	0.621	*–*1.073	1.368	0.237	0.813
**Austral season (winter): Sex (male)**	0.588	0.55	*–*0.492	1.667	1.069	0.285
***Austral season (spring): Sex (male)***	***1.452***	***0.533***	***0.406***	***2.498***	***2.726***	***0.007***
**Foraging rate: large items (df = 565)**						
**(Intercept)**	*–*2.853	0.33	*–*3.501	*–*2.205	*–*8.644	<0.001
***Austral season (autumn)***	***–1.833***	***0.363***	***–2.547***	***–1.119***	***–5.044***	***<0.001***
***Austral season (winter)***	***–3.732***	***0.317***	***–4.354***	***–3.11***	***–11.786***	***<0.001***
***Austral season (spring)***	***–1.566***	***0.308***	***–2.17***	***–0.962***	***–5.093***	***<0.001***

All models controlled for the individual identity of foxes. 95% confidence intervals (CI) are indicated as CI_low for the lower CI and CI_high for the higher CI. Significant effects are highlighted in bold and italicized text. For Austral season, summer was the reference, and female was the reference level for sex.

**Table 5:** Model outputs from a linear mixed effect model investigating variation in fecal glucocorticoid metabolites in wild bat-eared foxes.

Parameter	Coefficient	SE	CI_low	CI_high	*t*_324_	*P*
**(Intercept)**	–1.474	0.109	–1.689	–1.259	–13.489	<0.001
**Seasonal activity (Denning)**	0.273	0.158	–0.037	0.583	1.731	0.084
**Seasonal activity (Non-breeding)**	–0.054	0.139	–0.328	0.219	–0.392	0.696
***Parental status (Parent)***	**–*0.676***	***0.240***	**–*1.148***	**–*0.203***	**–*2.815***	***0.005***
***Seasonal activity (Denning): Parental status (Parent)***	***0.953***	***0.332***	***0.300***	***1.606***	***2.872***	***0.004***
***Seasonal activity (Non-breeding): Parental status (Parent)***	***0.688***	***0.329***	***0.041***	***1.335***	***2.093***	***0.037***

95% Confidence Intervals (CI) are indicated as CI_low for the lower CI and CI_high for the higher CI. Significant effects are highlighted as bold and italicized text. For seasonal activity, breeding season was the reference level, with non-parental status being the reference for parental status.

## Discussion

This study provides the first evidence of how foraging behavior and fGCM concentrations of bat-eared foxes change in parental individuals. Our first prediction, that foraging rates would be lowest in the environmentally challenging season of winter, was supported: all foxes showed lower consumption rates in this season. Male foxes exhibited a peak in foraging activity in spring, focusing on small items. Contrary to our second prediction, parenting did not impact foraging rates in this population. However, parenting did appear to affect fGCM levels, in line with our third prediction. Surprisingly, parental fGCM dropped significantly during the breeding season and showed no difference from fGCM in the general population during the denning season, when we expected parents to experience the highest relative stress. Parents appeared to respond similarly across all seasonal activities. Although based on a small sample size, these data suggest that bat-eared foxes respond well to the physiological demands of parental care despite the constraining factor of a very limited dietary niche.

Seasonal changes in prey availability—accompanied by significant variation in temperature and wind speeds (Lourens and Nel 1990; Renda et al. 2023)—did not appear to present significant challenges to bat-eared foxes. Although this species is a dietary specialist, habitat selection patterns of bat-eared foxes suggest that they do not closely follow the distribution of their primary prey, that is, harvester termites (*Hodotermes mossambicus*; Périquet and le Roux 2018). The decrease in foraging rates of all foxes during winter (similar to findings by Lourens and Nel 1990; Renda et al. 2023) could have been accompanied by glucocorticoid release to maintain homeostasis, but fGCM remained relatively constant across austral seasons. Such stability in fGCM mirrors the pattern in another socially monogamous small carnivore, the Aardwolf (*Proteles cristata*; Marneweck et al. 2015), but stands in contrast to a number of mammalian species that exhibit increases in fGCM during harsher seasons (Garber et al. 2020; Whipple et al. 2021). Our findings suggest that bat-eared foxes are not acutely challenged in a particular season, but that they cope behaviorally and physiologically with the relatively predictable changes in seasons. Their ability to find prey successfully despite the presence of strong winds (Renda et al. 2023) underscores the resilience of this specialist carnivore, who relies on its hearing to hunt (Renda and le Roux 2017).

It is notable that male foxes, regardless of parental status, sharply increase foraging rates in spring. Whereas increased foraging activity may activate the stress axis in some mammals (Zhang et al. 2020), there was no significant change in fGCM reflecting this heightened foraging behavior in male bat-eared foxes. We speculate that bachelor males would have been searching for mates during this period, requiring and finding more food during their forays, while successfully mated males could have been building up energy reserves before the potential appearance of pups. After pups are born, male bat-eared foxes babysit their pups at the den (Pauw 2000; Maas and MacDonald 2004; Wright 2006; Wright et al. 2010), similar to canids such as the Racoon Dog (*Nyctereutes procyonoides*), where fathers spend considerably more time at dens caring for their pups than their female counterparts (Malcolm 1986; Kauhala et al. 1998). We may have expected similar changes in foraging behavior in pregnant and lactating females, but with the small sample size of 1 mother, statistically significant patterns did not emerge. The monitored mother proved to be highly effective in hunting and consuming large food items such as a hare (reported by Jacobs and Le Roux 2016). She also caught the highest number of rodents (*n *= 35) in the population (le Roux et al. 2026). Given that the demands of nursing in bat-eared foxes often necessitate allocare (Pauw 2000) and may lead to the relinquishment of maternal care entirely (Ross and MacLarnon 2000), we suspect that her hunting skills and penchant for large mammalian prey are a behavioral mechanism to successfully meet the dietary needs of her maternal role.

The fGCM drop in parents during the breeding season potentially presents a challenge to the traditional idea (Romero and Beattie 2022) that cortisol increases in response to stressors—if we presume that breeding season is a time of higher stress for bat-eared foxes. The limited available evidence suggests that mammalian fathers in species with bi-parental care exhibit elevated glucocorticoids while their mates are pregnant (Saltzman and Ziegler 2014). Additionally, in carnivores like wolves (*Canis lupus*; Sands and Creel 2004), aggression and fGCM increase significantly during the mating period, which may not apply to bat-eared foxes, however. The fGCM patterns in bat-eared foxes do align with the traditional model if we consider that successful pairing, mating, and pregnancy in these monogamous foxes may be seen as not just an ultimate fitness boost, but also an immediate fitness benefit. Captive bat-eared foxes showed elevated fGCM after separation from their mates (le Roux et al. 2016), and general levels of social aggression are very low in this species (de Bruin et al. 2018). Aggression does not increase during mating season for bat-eared foxes; in this monogamous species, sexual competition appears to be low and mated pairs rarely copulate with extra-pair individuals (Wright et al. 2010). We therefore propose that changes in the social environment of bat-eared foxes—here, essentially, the formation of a small family group—have a stronger impact on their physiological stress-responses than do changes in the physical environment. Whether multi-year pair bonding and parenting with a long-term partner has a differential effect on foraging behavior and physiological stress remains to be determined.

## Supplementary Material

gyag011_Supplementary_Data

## Supplementary data

**Supplementary Data SD1**. Number of 2-h long observation sessions (focal follows) per individual per month from January (1) to December (12).

**Supplementary Data SD2**. Daily foraging rates for all bat-eared foxes.

**Supplementary Data SD3**. Daily foraging rates for small versus large items, for all bat-eared foxes.

**Supplementary Data SD4**. Fecal glucocorticoid concentrations for all bat-eared foxes.
