# Supplementary material for: Effect of reproductive status on foraging behavior and fecal glucocorticoid metabolite levels in wild bat-eared foxes (Otocyon megalotis)
Source: J Mammal. 2026 Mar 11;107(2):221–7. doi: 10.1093/jmammal/gyag011 (PMC13035264; doi:10.1093/jmammal/gyag011)
Supplement: gyag011_Supplementary_Data [file gyag011_supplementary_data.zip › Supplementary Data SD1.docx]

Supplementary Data SD1


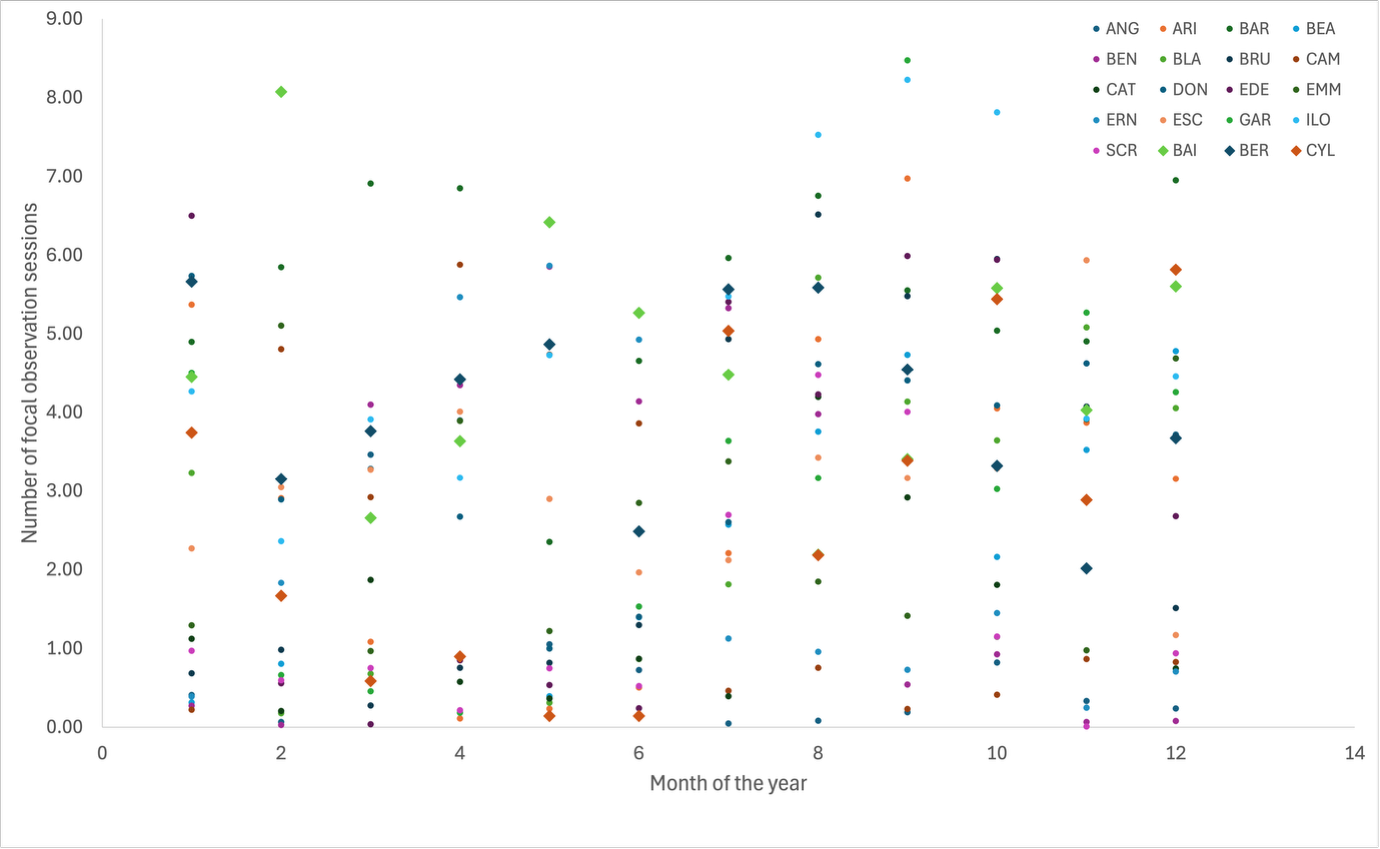


Figure S1. Number of 2-hour long observation sessions (focal follows) per individual per month from January (1) to December (12). Legend indicates individual code or identity. Parents are indicated by diamond shapes, while non-parents are indicated as smaller filled circles. Data are jittered slightly to improve visibility of each data point.
